# A systematic literature review of the clinical efficacy of repetitive transcranial magnetic stimulation (rTMS) in non-treatment resistant patients with major depressive disorder

**DOI:** 10.1186/s12888-018-1989-z

**Published:** 2019-01-08

**Authors:** Jeffrey Voigt, Linda Carpenter, Andrew Leuchter

**Affiliations:** 1 Medical Device Consultants of Ridgewood, LLC, 99 Glenwood Rd, Ridgewood, NJ 07450 USA; 2 Department of Psychiatry and Human Behavior, Brown Institute for Brain Science, Brown University, 700 Butler Dr, Providence, RI 02906 USA; 3 University of California Los Angeles (UCLA), Neuromodulation Division, Semel Institute for Neuroscience and Human Behavior, UCLA, 760 Westwood Plaza, Room 37-452, Los Angeles, CA 90095 USA

**Keywords:** Repetitive transcranial magnetic stimulation, Medication resistance, Clinical efficacy

## Abstract

**Background:**

The clinical efficacy of repetitive transcranial magnetic stimulation (rTMS) in treatment resistant patients (at least 4 medication trials) appears to be well accepted and forms the coverage policies and rTMS’s use in many of the largest US payers. However, less is known about rTMS’s use in patients who have undergone ≤1 failed medication trial. The purpose of this analysis was to determine the clinical efficacy of rTMS in patients after ≤1 medication trials.

**Methods:**

A systematic review of the literature was undertaken to identify all articles which addressed the use of rTMS in ≤1 medication trial. All types of study designs were included and assessed for quality and strength of evidence using: GRADE and CEBM. Searches of peer reviewed articles were undertaken for the year 2000 to the present. All languages were considered. Electronic databases were searched and included: PubMed and EBSCO. Evidence assessment websites were also searched and included: Cochrane, NICE, AHRQ, and ICER. Additionally, the clinical guidelines for specialty societies which use rTMS was searched. Hand searches of the reference sections of identified articles was also undertaken.

**Results:**

Electronic and other sources identified 165 after duplicates were removed. Twenty two articles were assessed for eligibility and ultimately 10 articles were included in the systematic review and graded. Six articles were graded high quality (CEBM/GRADE: 1c/B) demonstrating that the use of rTMS was clinically efficacious in patients after ≤1 medication trial. Four additional trials demonstrated a positive effect of rTMS in patients after ≤1 medication trial but were of a lower quality.

**Conclusion:**

The use of rTMS in patients after ≤1 medication trial should be considered. US payers should consider revising their coverage policies to include the use of rTMS in these patients.

**Electronic supplementary material:**

The online version of this article (10.1186/s12888-018-1989-z) contains supplementary material, which is available to authorized users.

## Background

Repetitive transcranial magnetic stimulation (rTMS) is an FDA cleared therapy for use in treating major depressive disorder (MDD). All products cleared for market use are indicated for: “*Treatment of major depressive disorder in adult patients who have failed to receive satisfactory improvement from prior antidepressant medication in the current episode* [1–5]*”.* The clinical efficacy of rTMS has been demonstrated in numerous randomized controlled clinical trials in patients who have failed 1–4 pharmacologic treatment regimens [6–8]. Additionally, medical specialty guidance documents support its use, including a whitepaper from the Clinical Transcranial Magnetic Stimulation (TMS) Society [9] and consensus recommendations published by a group of rTMS experts in the National Network of Depression Centers (NNDC) the American Psychiatric Association (APA) Council on Research [10].

Current medical policy coverage guidelines for the largest US payers call for coverage of rTMS if the following condition is met: “….*inability to tolerate psychopharmacologic agents (at least 3–4 trials of agents with distinct side effects)* [11–15]*”*. Unfortunately as patients move through the depression treatment paradigm they become more resistant to any therapy [16–19]. The American Psychiatric Council further states: “*A consistent predictor of antidepressant response across most therapeutic modalities is the degree of treatment resistance. Thus, rTMS is like other known antidepressant treatments in this respect with greater treatment resistance generally predicting poorer response* [10].*”*

Recently a local Medicare carrier has allowed for rTMS use after at most 1 failed pharmacologic therapy [20]. Additionally a lifetime cost effectiveness analysis examining the use of rTMS after one failed therapy and comparing it to standard therapy (i.e. multiple trials of pharmacologic agents) demonstrated that rTMS can be cost saving and improve upon the quality of life in the various age cohorts examined [21].

Based on the above, it is the purpose of this analysis to further examine the evidence on the use of rTMS in patients major depressive disorder who have failed ≤1 vs. ≥2 pharmacologic trials (as a comparison) to determine if there is clinical efficacy (and if clinical efficacy improves in patients who have undergone ≤1 failed medication trial vs. ≥2 failed medication trials [hereafter defined as treatment resistant]) when used after ≤1 failed medication trials. As well, it is the intention to examine patients who were treated with rTMS ± pharmacotherapy vs. pharmacotherapy as the first therapy in treatment naïve patients in order to determine rTMS’s clinical effect. This analysis appears not to have been done previously and may offer payers an alternative for cost savings and improved outcomes vs. numerous trials of pharmacologic agents.

## Methods

A systematic review of the literature was undertaken using the following sources and search terms:

Search terms: ((((Predict*) AND response) AND rTMS)) AND depress*. As well, search terms used were: ((((((rTMS) AND major) AND depress*) AND controlled) AND trial)) AND response.

Electronic searches were undertaken using both PubMed and EBSCO.

Other searches were made of the following websites:

- Technology Assessments: National Institute of Health and Clinical Excellence (NICE); Agency for Health Research and Quality (AHRQ); California Technology Assessment Forum (CTAF)/Institute for Clinical and Economic Review (ICER).
- Cochrane Database of Systematic Reviews
- Clinical consensus statements of specialty societies including the American Psychiatric Association and the Clinical TMS Society

Hand searches of the reference sections of all articles obtained were undertaken.

Articles which addressed the issue of the number of medication trials and rTMS outcomes in patients with MDD were included. More specifically, those trials which defined non-treatment resistant patients as ≤1 medication trial and evaluated rTMS outcomes were also included. Those trials that defined non-treatment resistance as ≤2 medication trials were excluded. The clinical outcome evaluated was clinical response to rTMS in treatment naïve or after 1 failed medication trial.

The level and quality of the evidence was assessed using the Center for Evidence Based Medicine (CEBM) [22] and Grading of Recommendations, Assessment, Development, and Evaluation (GRADE) [23] criteria. (See Additional file 1: Appendix 1 for the criteria used for each.)

Electronic and hand searches were performed by JV and adjudicated by LC. Assessment of the evidence was first performed by JV and confirmed by AL.

Lastly a Preferred Reporting Items for Systematic Reviews and Meta-Analyses (PRISMA) checklist was utilized to ensure the manuscript adhered to minimum accepted guidelines for systematic reviews (Additional file 2: Appendix 2).

## Results

### Electronic searches

Electronic databases were searched for the years: 2000 to the present. The year 2000 was chosen as rTMS began to be evaluated in patients about this time.

- PubMed searched on January 20, 2018 using the search terms: ((((Predict*) AND response) AND rTMS)) AND depress* - 91 hits; 10 records obtained
- PubMed searched on January 31, 2018 using the search terms: ((((((rTMS) AND major) AND depress*) AND controlled) AND trial)) AND response – 85 hits; 6 records identified; 3 duplicates; 2 new record obtained.
- EBSCO searched on January 21, 2018 using the search terms: ((((Predict*) AND response) AND rTMS)) AND depress* - 59 hits; 4 records identified; 3 duplicates; 1 new record obtained
- EBSCO searched on January 31, 2018 using the search terms: ((((((rTMS) AND major) AND depress*) AND controlled) AND trial)) AND response – 50 hits; 4 records identified. 4 duplicates; 0 new records obtained.

### Consensus recommendations by specialty societies

- American Psychiatric Association searched on January 20, 2018–1 hit; 1 record obtained
- Clinical TMS Society searched on January 20, 2018–1 hit; 1 record obtained

### Technology assessments/systematic reviews

- National Institute for Health and Clinical Excellence (NICE) searched on January 22, 2018–1 hit; 1 record obtained
- Agency for Health Research and Quality (AHRQ) Technology Assessments searched on January 22, 2018–1 hit; 1 record obtained
- Cochrane Database of Systematic Reviews; searched on January 23, 2018–1 hit; 1 record obtained.
- California Technology Assessment Forum (CTAF) searched on February 1, 2018–1 hit; 1 record obtained.

### Hand searches of reference sections of articles identified in above searches (searched on 1/23/18 & 1/31/18)

- Wang Y-M, et al. Randomized controlled trial of repetitive transcranial magnetic stimulation combined with paroxetine for the treatment of patients with first-episode major depressive disorder. Psych Research. 2017;254:18–23. 1 hit; 1 duplicate; 0 new records obtained.
- Wang H-N, et al. Clustered repetitive transcranial magnetic stimulation for the prevention of depressive relapse/recurrence: a randomized controlled trial. Trans Psych. 2017;7:1292. DOI 10.1038/s41398-0001-x. 1 hit; 1 new record obtained
- Huang M-L, et al. Repetitive transcranial magnetic stimulation in combination with citalopram in young patients with first-episode major depressive disorder: A double-blind, randomized sham-controlled trial. ANZJP. 2012;46(3):257–264. 2 hits; 2 duplicates; 0 new records found
- American Psychiatric Association. McClintock SM et al. Consensus recommendation for the clinical application of repetitive transcranial magnetic stimulation (rTMS) in the treatment of depression. Journal Clinical Psychiatry. 2017; doi.org/10.4088/JCP.16cs10905–5 hits; 5 new records obtained
- Perera T, et al. The clinical TMS society consensus review and treatment recommendations for TMS therapy for major depressive disorder. Brain Stimulation. 2016;9:336–346. – 4 hits; 3 duplicates; 1 new record obtained.
- Beuzon G, et al. Predictors of response to repetitive transcranial magnetic stimulation (rTMS) in the treatment of major depressive disorder. Encephalie. 2017;43:3–9. 2 hits that were duplicates; 0 new records obtained.
- Dumas R, et al. Stimulation magnétique trancrânienne répétée dans la prise en chage des épisodes dépressifs majeurs: facteurs prédictifs de response thérapeutique. L’Encéphale 2012;38:360–368. 5 hits that were duplicates; 0 new records obtained.
- Brakemeier E-L, et al. Patterns of response to repetitive transcranial magnetic stimulation (rTMS) in major depression: Replication study in drug-free patients. Journal Affect Disord. 2008;108:59–70. 2 hits; 2 duplicates found. 0 new records obtained.
- Brakemeier E-L, et al. Positive predictors for antidepressive response to prefrontal repetitive transcranial magnetic stimulation (rTMS). 2007. 41:395–403. 2 hits; 1 duplicate. 1 new record obtained.
- Fregni, F et al. Predictors of antidepressant response in clinical trials of transcranial magnetic stimulation. Inter Jrl Neuropsychopharm 2006;9:641–654. 1 hit; 1 new record found
- Carpenter LL, et al. Transcranial magnetic stimulation (TMS) for major depression: A multisite, naturalistic, observational study of acute treatment outcomes in clinical practice. Depress Anxiety 2012;29:587–596. 2 hits; 2 duplicates. 0 new records found
- O’Reardon JP et al. Efficacy and safety of transcranial magnetic stimulation in the acute treatment of major depression: A multisite randomized controlled trial. Biol. Psych. 2007;62:1208–1216. & Lisanby SH, et al. Daily left prefrontal repetitive transcranial magnetic stimulation in the acute treatment of major depression: Clinical predictors of outcome in a multisite, randomized controlled clinical trial. Neuropsychopharm. 2009;34:522–534. 3 hits; 3 duplicates. 0 new records found
- Mitchell PB, et al. Transcranial magnetic stimulation for depression. Austral New Zeal Jrl Psych. 2006;40:406–413. 0 hits
- Fitzgerald PB, et al. A study of the pattern of response to rTMS treatment in depression. Depress Anxiety. 2016;33:746–753. 4 hits; 2 duplicates. 2 new records obtained.
- Cohen RB, et al. Clinical predictors associated with duration of repetitive transcranial magnetic stimulation treatment for remission in bipolar depression. Jrl. Nerv Ment Disord. 2010;198:679–681. 1 hit; 1 duplicate. 0 new records found.
- Yang H, et al. A randomized controlled trial of right low frequency rTMS combined with escitalopram in treatment of patients with first-episode depression in general hospitals. JPBS. 2017;2(5):2. DOI: 10.20900/jpbs.20170016. 0 hits.

**Fig. 1:**
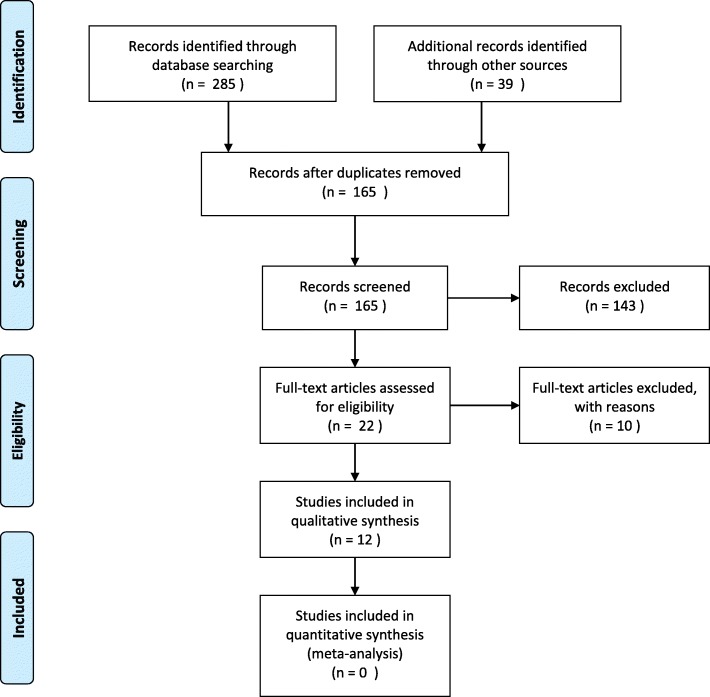
PRISMA Flow Diagram

Figure 1 depicts the flow diagram of articles screened, identified, eligible and excluded from the analysis. In total there were 10 articles identified which addressed the issue of rTMS efficacy based on the number of medication trials and which; focused on patients who were not defined as treatment resistant (≤1 medication failure), that a patient with MDD had undergone prior to use of rTMS. These studies are identified in Table 1. Table 2 shows those studies that were excluded with reasons.

**Table 1:** Studies included in the analysis

Study	Design	Baseline characteristics		Comparison # med trials (treatment resistance)	Outcome	CEBM/GRADE
Fregni F, et al. Inter Jrl Neuropsychophar. 2006 [24]	Pooled data from 6 clinical trials – retrospective analysis Countries: Canada (*N* = 25 patients), US (*N* = 60), Austria (*N* = 29), Brazil (*N* = 21)	153 patients; Age = 51.1 ± 15.1; M/F = 63/90		≥2 medication trials defined as refractory (medication resistant).	Treatment refractoriness was a significant predictor of clinical response to rTMS (*P* < 0.0001); use of Model 3 excluded Tel Aviv patients (*N* = 42) due to only one failed medication trial. Therefore 195–42 = 153 patients included in the analysis.	1c/B
Brakemeier E-L, et al. Jrl. Psych Res. 2007 [30]	Prospective case series Country: Germany	70 patients; Age = 49.5 ± 12.5; M/F = 44/26		Comparison medication resistant (≥2 trials) (*N* = 51) to non-medication resistant (1 trial) (*N* = 19)	Patients with a shorter duration of depressive episode and lower level of medication resistance showed a greater response to rTMS.	4/C
O’Reardon JP, et al. Biol Psych. 2007 [25]; Lisanby SH, et al. Neuropsychopharm. 2009 [18]	Double blind multisite (23 centers) RCT	301 patients; Age = 48.3 ± 10.8; M/F = 142/159		Comparison medication resistant (≥2 trials) (*N* = 147) to non-medication resistant (1 trial) (*N* = 164)	The likelihood of responding to rTMS was 4 times higher if patients had received one unsuccessful medication trial before rTMS in comparison with patients having received 2 or more unsuccessful trials (*P* = 0.021). Effect size in patients with one failed therapy was 0.83. Post hoc analysis performed by Lisanby.	1b/B
	Countries: 20 sites US; 2 Austria; 1 Canada					
Brakemeier E-L, et al. Jrl Affect Disord. 2008 [31]	Prospective and retrospective case series Country: Germany	79 patients; Age = 49.1 ± 14.3; M/F = 35/43		Comparison within rTMS treatment arm medication resistant (≥2 trials) to non-medication resistant (1 trial)	Non-treatment resistant patients with a short duration of episode were more likely to respond to rTMS than medication resistant (43% vs. 18%) (*P* = 0.023)	4/C
Cohen RB, et al. Jr. Nerv Ment Dis. 2010 [32]	Single center observation study Country: Brazil	56 patients; Age = 48 ± 15; M/F = 26/30		Comparison low treatment resistance [1 trial] (*n* = 34) to high treatment resistance [≥2 trials] (*N* = 22)	Low treatment resistance has a statistically significant effect (*P* < 0.01) on treatment outcome as measured by HDRS.	4/C
Carpenter LL, et al. Depress Anxiety. 2012 [33]	Multicenter observational study Country: US	307 patients; Age = 48.6 ± 14.2; M/F = 102/205		Comparison low treatment resistance [≤1 trial] (*n* = 140) to high treatment resistance [≥2 trials] (*N* = 167)	Low treatment resistance had a modest influence on treatment outcome as measured by CGI-S and PHQ-9 outcomes. No statistical difference between groups on response and remission but a higher percentage of patients having response (59.4% vs. 56.8%; CGI-S; 57.2% vs. 55.6%; PHQ-9) or remission (39.9% vs. 34.9%; CGI-S; 31.9% vs. 26%; PHQ-9)with low treatment resistance	4/C
Huang M-L, et al. Aust & NZ Jrl Psych. 2012 [26] (Note: Huang L et al., Zhejiang Da Xue Bio Yi Xue Ban. 2011 is a duplicate study [43]. However it is in Chinese so Huang M-L et al. Aust & NZ Jrl Psych 2012 used)	Single center RCT Country: China	Active = 28; Age = 32.8 ± 7.3; M/F = 9/19	Sham = 28; Age = 31.6 ± 7.4; M/F = 8/20	Comparison of rTMS plus citalopram (*N* = 28) vs. rTMS sham plus citalopram (N = 28) in first episode major depressive disorder on response and remission after 4 weeks. First 2 weeks use of rTMS (active or sham). Second two week citalopram only in both groups.	Significantly greater number of early improvers (using HAMD-17) at 2 weeks with rTMS vs. sham/citalopram (*P* = 0.031). No difference in response (46% vs. 36%; *P* = 0.586) or remission (39% vs. 29%; *P* = 0.572) at 4 weeks.	1b/B
Wang H-N, et al. Translational Psych. 2017 [27]	Single center RCT Country: China	rTMS+med = 82; Age = 42.3 ± 11.4;M/F = 22/60	Med =108; Age = 40 ± 11.5; M/F = 23/85	Comparison in first episode depressed patients: rTMS (*N* = 91) vs. antidepressant (*N* = 108) vs. rTMS plus antidepressant (*N* = 82) over 12 months. Examination of relapse/recurrence.	Relapse/recurrence at 12 months significantly lower in rTMS plus antidepressant group (20%) vs. antidepressant group (44.4%) (*P* = 0.033).	1b/B
Wang Y-M, et al. Psych Res. 2017 [28]	Single center RCT Country: China	Active = 22; Age = 28.8 ± 8.5; M/F = 12/10	Sham = 23; Age = 30.1 ± 9.5; M/F = 13/10	Comparison in treatment naïve patients rTMS plus paroxetine (N = 22) [active] to rTMS sham plus paroxetine (*N* = 21) [sham]	Response and remission rate of [active vs. sham] 95.5% vs. 71.4 and 68.2% and 38.1% respectively. (*P* < 0.05)	1b/B
Yang H, et al. Jrl Psych Brain Sci. 2017 [29]	Single center RCT Country: China	Active =41 patients; Age = 35.5 ± 12; M/F = 17/24	Sham = 41 patients; Age = 35.4 ± 12.1; M/F = 15/25	Comparison in treatment naïve patients rTMS plus escitalopram (*N* = 41) [active] to rTMS sham plus escitalopram (*N* = 41) [sham]	Active rTMS plus escitalopram significantly more effective (≥50% reduction in HAMD-17 score) (*N* = 36) than sham (*N* = 17) at 4 weeks (P < 0.05)	1b/B

**Table 2:** Studies excluded with reasons

Study	Reason excluded
Conca A, et al. Human Psychopharmacology. 2000 [44]	Did not examine effect of number of antidepressant trials on rTMS response
Cochrane Review. 2001 [45]	Review 17 years old. Did not examine effect on the number of antidepressant trials on rTMS response.
Holtzheimer PE, et al. Depression Anxiety. 2004 [46]	Patients treated with rTMS who responded/did not respond were identified as either having < 7 or > 7 antidepressant trials.
Mitchell PB, et al. Austral New Zeal Jrl Psych. 2006 [34]	Descriptive review of 25 rTMS studies. Stated that patients who were more treatment resistant (resistance not defined) were less likely to respond to rTMS.
CTAF, 2009 [47]	Review 8 years old. However did reference one study already included in assessement [18].
AHRQ. 2011 [35]	Did not examine number of failed medication trials effect on rTMS
Aguirre AK, et al. Jrl Affective Disord. 2011 [48]	Age only was examined as a predictor of rTMS efficacy.
Fitzgerald PB, et al. Expert Reviews 2011[49]	Stated patients were not treatment resistant. However, in examining paper, patients were found to have at least 2 failed medication trials.
Connolly KR, et al. Jrl, Clinical Psych 2012 [40]	Jrl Clin Psych 2017 rTMS consensus recommendations [10] stated there was no relationship between degree of treatment resistance and response to rTMS in this study. However, in this case series analysis it was found that patients were had an average of 3.4 failed medication trials and were found to be treatment resistant - with no direct comparison group.
NICE 2015 [36]	Did not examine number of failed medication trials effect on rTMS
Levkovitz Y, et al. World Psych. 2015 [8]	Multicenter (20 centers) RCT. Countries: 14 sites US, 4 Israel, Germany, and 1 Canada. Total of 212 patients (ITT), 181 patients (Per protocol). Comparison low medication treatment resistance (≤2 trials) to ≥3 failed treatments. Patients treated with rTMS who failed ≤2 treatments significantly more responsive (*P* = 0.032) than those with ≥3 treatments (*P* = 0.057).
Fitzgerald PB, et al. Depression Anxiety. 2016 [50]	Patients treated with rTMS who responded/did not respond had on average 5.7–6.1 failed medication trials. Could not break out low vs. high medication treatment resistance.

As can be seen in Table 1, there were six studies that would be considered of high quality as evaluated by CEBM/GRADE criteria which demonstrated a statistically significant and positive effect of rTMS in patients with low medication resistance (≤1 trial) prior [24–29]. The other trials were of lower quality but again, all demonstrated a positive effect of rTMS in patients with low medication resistance (≤ 1 trial) [30–33]^,^. Two studies related to treatment resistance were excluded for the following reasons: Lefkovitz [8] was excluded as it compared ≤2 medication trials to ≥3 trials. It did however find that patients treated with rTMS who failed ≤2 medication trials were significantly more responsive (*P* = 0.032) vs. those with ≥3 medication trials. Mitchell [34] was excluded as it did not specify the number of medication trials but did state that the number of medication trials affected rTMS’s efficacy.

Adverse events (where identified) included Table 3 and mainly consisted of headache and scalp pain [24, 25, 27–29, 32]. These adverse events were transitory in nature.

**Table 3:** Adverse events

Study	Reported adverse events
Fregni F, et al. Inter Jrl Neuropsychopharm. 2006 [24]	*N* = 54; Included headache, neck pain and scalp burn
Brakemeier E-L, et al. Jrl. Psych Res. 2007 [30]	Not a defined endpoint.
O’Reardon JP, et al. Biol Psych. 2007 [25]; Lisanby SH, et al. Neuropsychopharm. 2009 [18]	Active rTMS: eye pain (*n* = 10); GI & toothache (*n* = 12); site discomfort (*n* = 18); site pain (*n* = 59); facial pain (*n* = 11); muscle twitching (*n* = 334); pain of skin (*n* = 14).
	Sham: eye pain (n = 3); GI & toothache (n = 1); site discomfort (*n* = 2); site pain (*n* = 6); facial pain (*n* = 5); muscle twitching (*n* = 5); pain of skin (*n *= 1)
Brakemeier E-L, et al. Jrl Affect Disord. 2008 [31]	Not a defined endpoint
Cohen RB, et al. Jr. Nerv Ment Dis. 2010 [32]	Headache (*n* = 6); increased somnolence (*n* = 2); nightmares (*n* = 3)
Carpenter LL, et al. Depress Anxiety. 2012 [33]	Tonic/clonic seizure (*n* = 1)
Huang M-L, et al. Aust & NZ Jrl Psych. 2012 [26] (Note: Huang L et al., Zhejiang Da Xue Bio Yi Xue Ban. 2011 is a duplicate study [43]. However it is in Chinese so Huang M-L et al. Aust & NZ Jrl Psych 2012 used)	Not a defined endpoint
Wang H-N, et al. Translational Psych. 2017 [27]	rTMS + meds: diarrhea (*n* = 5); constipation (*n* = 28); dry mouth (*n* = 43); nausea (*n* = 3); palpitations (*n* = 11); dizziness (*n* = 8); headache (*n* = 6); blurred vision (*n* = 21); tinnitus (*n* = 14).
	Meds: diarrhea (*n* = 8); constipation (*n* = 35); dry mouth (*n* = 66); nausea (*n* = 8); palpitations (*n* = 9); dizziness (*n* = 8); headache (n = 2); blurred vision (*n* = 15); tinnitus (*n* = 3).
Wang Y-M, et al. Psych Res. 2017 [28]	Active rTMS: headache/scalp pain (*n* = 7); Sham: headache/scalp pain (*n* = 8)
Yang H, et al. Jrl Psych Brain Sci. 2017 [29]	Active rTMS: scalp pain & dizziness (*n* = 2)

Based on the heterogeneity of the included studies, a further breakdown of the clinical response to rTMS was undertaken based on the number of medication trials prior to its use (Table 4). What can be seen in Table 4 are the following findings: the lower the number of medication trials, the better the response rate to rTMS; in patients with a ≤ 1 medication trial, the use of rTMS plus medication resulted in a response that was significantly higher vs. medication only; and in patients with ≤1 medication trial vs. ≥2 medication trials the use of rTMS provided an improved response.

**Table 4:** Breakout of studies based on number of medication trials prior to rTMS use

Number of medication trials	Studies	Comparator	Outcomes/effect; study duration
≥2 medication trials	Fregni F, et al. (2006) [24]		Higher response rate to rTMS therapy in patients who had a lower number of refractory treatment trials.
≤1 medication trial	Huang M-L, et al. (2012) [26];	rTMS plus med vs. sham plus med	Significantly higher number of improvers at 4 weeks with rTMS plus med.
	Wang H-N, et al. (2017) [27];	rTMS plus med vs. med	Significantly higher number in response and remission at 12 months with rTMS plus med.
	Wang Y-M, et al. (2017) [28];	rTMS plus med vs. sham plus med	Response and remission significantly higher in the rTMS + med
	Yang H, et al. (2017) [29]	rTMS plus med vs. sham plus med	Response and remission significantly higher in the rTMS + med
≥2 medication trials vs. ≤1 medication trial	Brakemeier E-L, et al. (2007) [30];	Use of rTMS with Low (1 trial) vs. high (≥2 trials) medication treatment resistance	Likelihood of response was higher in low treatment resistant patients
	O’Reardon JP, et al. (2009) [25];	Use of rTMS with Low (1 trial) vs. high (≥2 trials) medication treatment resistance	Likelihood of response was 4X higher and statistically significantly different in low treatment resistant patients
	Brakemeier E-L, et al. (2008) [31];	Use of rTMS with Low (1 trial) vs. high (≥2 trials) medication treatment resistance	Use of rTMS in low treatment resistant patients had a statistically significant effect on improved outcomes
	Cohen RB, et al. (2010) [32];	Use of rTMS with Low (1 trial) vs. high (≥2 trials) medication treatment resistance	Use of rTMS in low treatment resistant patients had a statistically significant effect on improved outcomes
	Carpenter LL, et al. (2012); [33]	Use of rTMS with Low (≤1 trial) vs. high (≥2 trials) medication treatment resistance	Use of rTMS in low treatment resistant patients had a modest effect on improving outcomes.

## Discussion

We present results of the first systematic examination of published clinical trial data to specifically demonstrate that the use of rTMS therapy in patients with ≤1 failed medication trials produces superior outcomes compared to those observed in patients who exhibit higher levels of medication resistance. It is known that the use of rTMS in treating MDD has demonstrated clinical efficacy in high quality studies and in patients who have previously failed 1–4 medication trials [35, 36]. This systematic review/analysis extends the understanding of the scope of rTMS’ therapeutic potential, and identified several clinical trials which show improved clinical efficacy of rTMS when used in depressed patients characterized by less pharmacoresistance (≤1 medication trials). The effect of increased treatment resistance in patients as medication trials increase is also a consistent finding with other therapeutic modalities [16–19]. It is also known that 20–40% of patients do not benefit from, or cannot tolerate adverse effects from, serial adequate trials of antidepressant medications [37]. It is thus important to identify treatments that can provide clinical benefit to the patient as early on as possible.

In a recent cost effective analysis, it has been found that the introduction of rTMS therapy after one failed medication therapy may cost less and provide for similar or even better outcomes when compared with serial medication trials over the life of the patient [21]. The findings from this cost effectiveness analysis are in line with other cost effectiveness analyses which examined patients over 9 weeks [38], and 3 years [39]. However, the main methodological difference between these prior reports and that of the Voigt et al. analysis [21] is the examination of cost effectiveness in patients who are not treatment resistant patients (i.e. after only one failed therapy). As rTMS is more clinically efficacious in patients who have failed ≤1 failed medication trial, and the likelihood that it will cost less in a less pharmacoresistant population, may be reason for payers to re-think coverage policies which restrict rTMS coverage to only depressed patients who present after 4 failed medication trials. The present findings support consideration of rTMS coverage after only one failed medication therapy, consistent with at least one Medicare local coverage determination policy which covers rTMS services for appropriate candidates after only one failed antidepressant medication therapy [20].

The 2016 Clinical TMS Society Consensus review of rTMS for MDD did not address the issue of treatment resistance [9]. While the results of the present analysis are in agreement with comments in a recent consensus recommendations paper [10], the current analysis differs with regard to the conclusions summarized by McClintock et al. [10]. They included data from a large, multisite, an open case series which was excluded from the current analysis due to the fact that it did not break out treatment resistance [40]. Additionally, further scrutiny of a large naturalistic study [33] that shaped the general conclusions of McClintock et al. [10] shows that while there was not a statistically significant difference in outcomes between non-treatment/treatment resistant subgroups, there was also an identified trend favoring the clinical efficacy of rTMS in patients who have undergone ≤1 failed medication trial.

While a direct comparison of response and remission rates based on degree of treatment resistance cannot be made, comparing response and remission rates from different studies may provide some insights. As it relates to the response and remission rates after failed therapies for both medication and rTMS, the literature shows that with medication the remission and response rates were: 30.6 and 28.5% after one failed medication therapy; 13.7 and 16.8% after 2 failed medication therapies and; 13 and 16.3% after 3 failed medication therapies [41]. The response and remission rates for rTMS were noted to be as follows: 95% response and 63% remission rate in treatment naïve patients [28]; 43% response rate after one failed medication trial [31]; 36.6% remission after one to two failed medication therapies [18] and; 28.9% remission after 3–4 failed medication therapies [18]. The types of patients in each of these studies appear to be comparable when evaluating the baseline characteristics [18, 31, 41]. Based on the data, it appears rTMS may provide at least comparable remission and response outcomes to antidepressant pharmacotherapy, based on treatment resistance.

Lastly, based on the results in Table 4, there appears to be a durable and improved response to the use of rTMS plus medication vs. medication only in patients who have failed ≤1 medication trials. These trials were well designed (i.e. RCTs). The use of rTMS as augmentative therapy to medication in treatment resistant patients (≥2 medication trials) has also demonstrated an improved response in a systematic review and meta-analysis [42]. The fact that similar results are demonstrated in patients who have failed ≤1 failed medication trial likely holds promise for rTMS as augmentative therapy in these types of patients.

## Limitations

The use of PubMed, EBSCO, and English language journals may have missed non-English language publications. The risk of bias in each study was not assessed. However, CEBM and GRADE assessments were evaluated. Four of the studies identified were randomized controlled trials [27–29, 43]. In each of these trials it was identified that the GRADE quality of evidence was moderate (level B).

## Conclusion

High quality evidence exists supporting the clinical efficacy of rTMS in patients who have failed ≤1 medication therapies. This evidence also appears to be in line with remission and response rates of patients who have undergone additional medication trials after one failed medication trial. High quality evidence also exists that rTMS used solely or in combination with antidepressants for first-episode major depressive disorder may be more effective than antidepressants alone. Thus the use of rTMS may shorten the treatment odyssey for patients with MDD. Further, cost-effectiveness has been demonstrated in patients with one failed rTMS therapy. Lastly, payers are beginning to cover rTMS after one failed medication trial – with one Medicare payer out of 7 doing so - Novitas [20]. Private payers in the US are not. Therefore rTMS should be considered for coverage with patients who have failed ≤1 failed medication trials.

## Additional files

Additional file 1: **Appendix 1.** – CEBM and GRADE. (DOCX 32 kb)

Additional file 2: **Appendix 2.** PRISMA checklist. (DOC 114 kb)

## Abbreviations

AHRQ: Agency for Healthcare Research and Quality
APA: American Psychiatric Association
CEBM: Centre for Evidence Based Medicine
CTAF: California Technology Assessment Forum
EBSCO: Elton B. Stephens Company
GRADE: Grading of Recommendations, Assessment, Development, and Evaluation
ICER: Institute for Clinical and Economic Review
NICE: National Institute for Health and Clinical Excellence
NNDC: National Network of Depression Centers
PRISMA: Preferred Reporting Items for Systematic Reviews and Meta-Analyses
rTMS: repetitive transcranial magnetic stimulation
TMS: Transcranial magnetic stimulation
