# Supplementary material for: A systematic literature review of the clinical efficacy of repetitive transcranial magnetic stimulation (rTMS) in non-treatment resistant patients with major depressive disorder
Source: BMC Psychiatry. 2019 Jan 8;19:13. doi: 10.1186/s12888-018-1989-z (PMC6325728; doi:10.1186/s12888-018-1989-z)
Supplement: Supplementary file 1 — Appendix 1. – CEBM and GRADE. (DOCX 32 kb) [file 12888_2018_1989_MOESM1_ESM.docx]

**Additional file 1: Appendix 1.**

Center for Evidence Bases Medicine (CEBM) criteria:

| **1a:** | Systematic reviews (with homogeneity) of randomized controlled trials |
| --- | --- |
| **1b:** | Individual randomized controlled trials |
| **1c:** | All or none randomized controlled trials |
| **2a:** | Systematic reviews (with homogeneity) of cohort [a group of people with defined characteristics who are followed up on based on an intervention to determine morbidity or mortality from a specified outcome(s)] studies |
| **2b:** | Individual cohort study or low quality randomized controlled trials (e.g. <80% follow-up) |
| **2c:** | "Outcomes" Research; ecological studies |
| **3a:** | Systematic review (with homogeneity) of case-control studies. |
| **3b:** | Individual case-control study |
| **4:** | Case-series (and poor quality cohort and case-control studies) |
| **5:** | Expert opinion without explicit critical appraisal, or based on physiology, bench research or "first principles" |

Grading of Recommendations Assessment, Development and Evaluation (GRADE)

| **Code** | **Quality of Evidence** | **Definition** |
| --- | --- | --- |
| A | High | Further research is very unlikely to change our confidence in the estimate of effect.   - Several high-quality studies with consistent results - In special cases: one large, high-quality multi-centre trial |
| B | Moderate | Further research is likely to have an important impact on our confidence in the estimate of effect and may change the estimate.   - One high-quality study - Several studies with some limitations |
| C | Low | Further research is very likely to have an important impact on our confidence in the estimate of effect and is likely to change the estimate.   - One or more studies with severe limitations |
| D | Very Low | Any estimate of effect is very uncertain.   - Expert opinion - No direct research evidence - One or more studies with very severe limitations |

Adapted from the Cochrane Handbook. Higgins, J.P.T., Green, S. (2011). Cochrane Handbook for Systematic Reviews of Interventions Version 5.1.0 [updated March 2011]. *The Cochrane Collaboration*, 2011. Retrieved 3/5/17 from: www.handbook.cochrane.org.
